# Editorial: Advances in anthocyanins: Sources, preparation, analysis methods, bioavailability, physiochemical properties, and structural features

**DOI:** 10.3389/fnut.2023.1148051

**Published:** 2023-02-16

**Authors:** Wuyang Huang, Zhongquan Sui, Weimin Zhang

**Affiliations:** ^1^Institute of Agro-Product Processing, Jiangsu Academy of Agricultural Sciences, Nanjing, China; ^2^School of Food and Biological Engineering, Jiangsu University, Zhenjiang, China; ^3^Department of Food Science and Technology, School of Agriculture and Biology, Shanghai Jiao Tong University, Shanghai, China; ^4^School of Food Science, Hainan University, Haikou, Hainan, China

**Keywords:** bioavailability, stability, products, preparation, anthocyanins, processing

Anthocyanins are compounds belonging to the class of flavonoids, characterized by their red-blue color, and exist extensively in plants, such as fruits, vegetables, and flowers including berries (blueberries, red currant, black currant, blackberries, cranberries, cowberries, lingonberries, raspberries, bilberries, cloudberries, gooseberries, rowanberries, and strawberries), edible flowers (pansies and snapdragons), purple carrots, and purple potatoes. These molecules have attracted much interests for many years, attributing to their possible therapeutic and beneficial effects. Anthocyanins can not only be used as natural colorants but also be potentially valuable for reducing coronary heart disease, preventing cancer/tumor, inflammatory and diabetic symptoms, as well as the improvement of visual acuity and cognitive behavior (1).

However, the chemical instability of anthocyanins probably results in their poor bioavailability and seriously limits their application in food and medical industries. These compounds are susceptible to degrade and significantly lose color due to several environmental factors, including temperature, light, oxygen, enzymes, and pH (2). In *in vivo* studies, the bioavailability of anthocyanins has been estimated to be merely around 0.26–1.8% (3). Thus, exploring how to increase the stabilization of anthocyanins has very important significance.

Different novel delivery systems, such as nanoencapsulation, microencapsulation, and protein complexes, have been developed and proposed in recent years to enhance the anthocyanins' bioavailability (4). Moreover, the high protein or high fat in food matrices may also give a protective effect to anthocyanins or even affect their bioavailability. The combination of protein with anthocyanins is a useful alternative for attenuating the degradation of anthocyanins during digestion and thus promoting the concentration of metabolites in plasma (5).

This Research Topic is aimed at collecting papers suitable to improve our knowledge and understanding on the sources, preparation, purification, analysis methods, bioavailability, stability, processing, physiochemical properties, and structural features of anthocyanins. There are four papers covering the above-mentioned aspects in this special e-collection.

As well known, the instability and non-targeted release of anthocyanins is becoming a major obstacle to realize their biological benefits in food systems. In order to improve the bioavailability of anthocyanins, numerous encapsulation approaches have been developed for the targeted release of anthocyanins while retaining their bioactivities. Natural biopolymers-based systems, including proteins and carbohydrates, can be designed for anthocyanins encapsulation. Song et al. systematically reviewed and highlighted the factors involved in anthocyanin stability, the properties and performance of protein- or/and polysaccharide-based encapsulation. The current understanding and challenges of biopolymers-based anthocyanins encapsulation are summarized in detail in this review paper. The proposed perspective can provide new insights into the improvement of anthocyanin bioavailability by edible biopolymer encapsulation, as well as the application of anthocyanins in food products. Similarly, during the protein gelation, the salt concentration is a common means to modify the morphology of protein aggregates and thereby control the product properties in food industry. Niu et al. investigated the influence of KCl concentration on the gelation of myofibrillar protein giant squid (*Dosidicus gigas*), which could shed light on food development with the guide of interactions between components to achieve the desired quality of products.

Moreover, the processing method exerts an influence on the physicochemical and functional properties of bioactive compounds. There are two papers on processing. Wei et al. studied on the influences of drying treatment. So as for anthocyanins, the industrial processing, involving steps of blanching, pasteurization, etc. decreased their content in the final products. The conventional solid-liquid extraction, followed by the effective adsorption using microporous resins is usually applied among the main technologies to utilize in depth the anthocyanins from plant materials. By optimizing the successive processes of extraction and purification (Wu et al.), the anthocyanin extracts from blackberry contained nine main pigments, which are divided into three aglycone-based forms and cyanidin-3-*O*-glucoside is the most abundant among them. The blackberry purified anthocyanin extracts show much higher antioxidant and antibacterial activities, cytoprotective effects, digestive enzyme inhibition than the crude anthocyanin extracts.

In summary, the above-mentioned studies and reviews represent high quality advance research and knowledge contributed by various research groups of the world working on anthocyanins. Despite all the existing literature and evidence related to this extremely important topic, the papers published in this special e-collection clearly show that there are still many aspects to be clarified and understood in anthocyanins.

## Author contributions

WH wrote the introduction and the conclusion. ZS and WZ wrote the central part with comments to the cited papers and references. All authors contributed to the article and approved the submitted version.
